# Iron isotopic fractionation between silicate mantle and metallic core at high pressure

**DOI:** 10.1038/ncomms14377

**Published:** 2017-02-20

**Authors:** Jin Liu, Nicolas Dauphas, Mathieu Roskosz, Michael Y. Hu, Hong Yang, Wenli Bi, Jiyong Zhao, Esen E. Alp, Justin Y. Hu, Jung-Fu Lin

**Affiliations:** 1 Department of Geological Sciences, Jackson School of Geosciences, University of Texas at Austin, Austin, Texas 78712, USA; 2 Origins Laboratory, Department of the Geophysical Sciences and Enrico Fermi Institute, The University of Chicago, 5734 South Ellis Avenue, Chicago, Illinois 60637, USA; 3 IMPMC–UMR CNRS 7590, Sorbonne Universités, UPMC, IRD, MNHN, Muséum National d'Histoire Naturelle, 61 rue Buffon, 75005 Paris, France; 4 Advanced Photon Source, Argonne National Laboratory, Argonne, Illinois 60439, USA; 5 Center for High Pressure Science and Technology Advanced Research (HPSTAR), Pudong, Shanghai 201203, China; 6 Department of Geology, University of Illinois at Urbana-Champaign, Urbana, Illinois 61801, USA

## Abstract

The +0.1‰ elevated ^56^Fe/^54^Fe ratio of terrestrial basalts relative to chondrites was proposed to be a fingerprint of core-mantle segregation. However, the extent of iron isotopic fractionation between molten metal and silicate under high pressure–temperature conditions is poorly known. Here we show that iron forms chemical bonds of similar strengths in basaltic glasses and iron-rich alloys, even at high pressure. From the measured mean force constants of iron bonds, we calculate an equilibrium iron isotope fractionation between silicate and iron under core formation conditions in Earth of ∼0–0.02‰, which is small relative to the +0.1‰ shift of terrestrial basalts. This result is unaffected by small amounts of nickel and candidate core-forming light elements, as the isotopic shifts associated with such alloying are small. This study suggests that the variability in iron isotopic composition in planetary objects cannot be due to core formation.

## Abstract (web summary)

Terrestrial basalts have a unique iron isotopic signature taken as fingerprints of core formation. Here, high pressure studies show that force constants of iron bonds increase with pressure similarly for silicate and metals suggesting interplanetary isotopic variability is not due to core formation.

Iron, as one of the most abundant non-volatile elements in the Solar System, plays a major role in every stage of planetary formation and differentiation. Much information can be gained on these processes by studying iron isotopic variations^1^,^2^,^3^,^4^,^5^,^6^,^7^,^8^. A striking feature of the Earth is the manner in which it is stratified into a core dominated by metallic Fe^0^, a mantle that is rich in ferrous Fe^2+^, and surface oxidative environments where iron is found mostly as ferric Fe^3+^ (ref. 9). Understanding how this stratification was established has important bearing on several questions in Earth and planetary sciences, such as the nature of the material accreted by the Earth as a function of time^10^,^11^ and the cause for the Great Oxygenation Event^12^. The abundance and redox state of iron in Earth's mantle reflects the redox conditions that prevailed during and after core formation^13^,^14^. Iron isotopes add a new dimension to this system but progress in interpreting Fe isotope variations has been hampered by the paucity of equilibrium isotopic fractionation factors under the *P–T* conditions relevant to core formation.

Mid-ocean ridge basalts (MORBs), which constitute the bulk of Earth's oceanic crust, are enriched in the heavy isotopes of iron by ∼+0.1‰ relative to chondrites (ref. 6; δ^56^Fe is the deviation in permil of the ^56^Fe/^54^Fe ratio relative to the IRMM-014 standard, whose composition is indistinguishable from chondrites). In contrast, basalts from Mars and Vesta have the same δ^56^Fe value as chondrites within uncertainty^2^,^4^,^5^,^15^,^16^. The non-chondritic Fe isotope composition of terrestrial basalts has been interpreted as resulting from iron vaporization into space during the Moon-forming giant impact^2^, high-temperature and high-pressure equilibrium fractionation between metal and silicate during core formation^7^,^8^, or iron isotopic fractionation during partial mantle melting to produce Earth's crust^17^,^18^,^19^,^20^. Supporting the last explanation is the observation that mantle peridotites have an iron isotopic composition that is close to that of chondrites^17^,^19^. However, mantle peridotites are imperfect proxies of the mantle composition, as they have been affected by partial melting and metasomatism^17^,^21^,^22^, which can fractionate Fe isotopes.

To test the core-mantle segregation mechanism and set constraints on the conditions of core formation, it is critical to know how iron isotopes are partitioned at equilibrium between metal and silicate under conditions relevant to core formation. Previous studies have investigated metal-silicate Fe isotopic fractionation at high temperature and relatively low pressure, reaching the conclusion that the fractionation would be negligible^23^,^24^. However, pressure is an important intensive parameter that affects the strength of iron bonds and the structure of silicates, so iron isotopic fractionation between silicate and metal might differ at high pressure^7^,^25^.

Polyakov^7^ used previously published results from nuclear resonant inelastic X-ray scattering spectroscopy (NRIXS) to conclude that the equilibrium iron isotopic fractionation between metallic iron and silicate post-perovskite under Earth's core-mantle boundary conditions may be sufficiently large to impart a measurable isotopic shift to the mantle. The NRIXS data used to support this claim are of insufficient precision at the low- and high-energy ends of the spectrum to derive reliable force constant values^20^. Furthermore, silicate post-perovskite would not be stable in the hot lowermost mantle of early Earth^26^ and its role in setting the present Fe isotopic signature of MORBs is uncertain.

Shahar *et al*.^25^ investigated iron isotope fractionation between bridgmanite and iron alloys (FeO, FeH_*x*_ and Fe_3_C) at pressures relevant to core formation in a typical magma ocean setting, up to ∼60 GPa, by using NRIXS spectroscopy complemented by theoretical calculations. They found that the effect of pressure on iron isotopic composition is different among iron-light element alloys investigated. Notably, the δ^56^Fe value of the current terrestrial mantle would be fractionated by ∼+0.02‰ relative to the bulk Earth composition for a pure iron core and this fractionation would increase to ∼+0.04‰ for an FeH_*x*_ or Fe_3_C core. Hydrogen and carbon are, however, unlikely major light element candidates in Earth's core^27^,^28^. Oxygen is more probable but Shahar *et al*.^25^ showed that it would induce a limited shift in the Fe isotopic composition of the mantle (∼+0.02‰).

The studies done so far have focused on crystalline phases as analogues to the magma ocean silicate melt^7^,^25^. Furthermore, the effects of Si, S (two of the major light element candidates in the Earth's core) and Ni alloying with Fe on iron isotopic fractionation remain uncertain. Therefore, it is crucial to examine iron isotopic fractionation between silicate glasses of basaltic composition and iron-rich alloys of Fe-Ni-Si, Fe-Si and Fe-S, to evaluate whether core formation could have fractionated the iron isotopic composition of Earth's mantle.

Here we report the experimental determination of the mean force constant 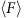 of iron bonds in synthetic samples of basaltic glass, metallic iron and iron-rich alloys of Fe-Ni-Si, Fe-Si and Fe-S up to 206 GPa. We find that all 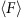 values increase with pressure, and that the 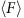 values of silicate glass are comparable to those of metal. Our results suggest that the interplanetary variability in iron isotopic composition cannot be due to core formation.

## Results

### Experimental approach

We studied the high-pressure equilibrium Fe isotopic fractionation between basaltic glass and Fe-rich Fe-Ni-Si, Fe-Si and Fe-S solid alloys (taken as proxies for molten silicates^29^,^30^,^31^ and alloys, respectively) using NRIXS spectroscopy with samples loaded in diamond anvil cells (DACs)^7^,^32^. NRIXS gives the mean force constant of iron bonds 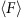, from which reduced partition function ratios or *β*-factors can be deduced through^32^,


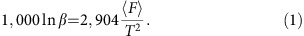


The *β*-factors give the extent to which ^56^Fe/^54^Fe ratios are fractionated between coexisting phases at equilibrium,


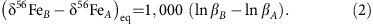


The starting samples were a fully reduced ^57^Fe-enriched basaltic glass containing only Fe^2+^ (ref. 20), polycrystalline Fe and Fe_86.8_Ni_8.6_Si_4.6_, Fe_85_Si_15_ and Fe_3_S alloys^33^,^34^,^35^. Conventional Mössbauer spectroscopic data, electron microprobe analyses and NRIXS data collected on the basaltic glass at ambient conditions were reported elsewhere^20^. Metal iron and the iron-based alloys were examined by X-ray diffraction and most were in a body-centred cubic structure at ambient conditions, except for Fe_3_S, which was in the tetragonal structure. The reported 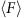 values for Fe_92_Ni_8_, Fe_85_Si_15_ and Fe_3_S are based on newly acquired NRIXS data combined with previous data from Lin *et al*.^34^,^35^, who did not report the 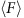 values for Fe_92_Ni_8_ and Fe_85_Si_15_. For previously acquired data, the uncertainties on the 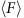 values are large due to the relatively narrow energy scans acquired, as those studies focused on the determination of the Debye sound velocity for seismological applications.

Although the phases relevant to core formation conditions are molten, direct measurements of vibrational properties on silicate and metallic liquids under ultrahigh pressure conditions are currently beyond experimental capabilities. Still, the behaviours of silicate melts under high *P–T* conditions can be understood by examining silicate glasses, because both exhibit similar microscopic and macroscopic properties^29^,^30^. For instance, the inelastic vibrational properties of silicate glasses are close to their molten counterparts^36^ and changes in the Si–O coordination number of molten basalt are in excellent agreement with those observed in silica glass at pressures up to 60 GPa (ref. 37). Similarly, for lack of better alternatives, crystalline iron is often used as proxy for molten iron in deep Earth studies. Molten iron is described as a simple close-packed liquid^38^ and exhibits comparable density and compressibility relative to its solid phase under core conditions^39^. As a first pass on this question and following Polyakov^7^ and Shahar *et al*.^25^, we take the 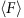 values of iron bonds in solid Fe and Fe-rich alloys to be proxies of those in their liquid counterparts. We use new data for the most relevant candidate light elements Si and S, and for the main alloying element Ni, together with literature data for FeH_*x*_, Fe_3_C and FeO alloys from Shahar *et al*.^25^, to fully evaluate how alloying and light elements affect iron *β*-factors of pure iron under high-*P*, *T* conditions.

### High-*T* harmonic extrapolation

Measurements of the force constant of iron bonds at room temperature were used to calculate the iron *β*-factors using equation (1), which assumes harmonic behaviour^7^,^20^,^25^,^32^,^40^ (see Methods). At the high temperatures relevant to core formation on Earth, the iron *β*-factors could be affected by anharmonic effects, but such effects may be largely suppressed by the associated high pressures^7^. Furthermore, theoretical caculations^25^,^41^,^42^,^43^ support the validity of the harmonic approximation for calculation of *β*-factors at high temperatures. For instance, the correction on the iron *β*-factors between 0 K and mantle temperatures is thought to be negligible^25^. NRIXS predictions have previously been shown to be in agreement with independent determination of equilibrium fractionation factors based on mass spectrometry analyses of coexisting phases thought to have achieved equilibrium. This is the case for magnetite and olivine at 873–1,073 K (refs 40, 44). This is also the case for basaltic glass and iron, for which NRIXS data^20^ agree with equilibration experiments performed in the molten silicate-metal system at 1,573 K and 1 GPa (ref. 24), and 2,273 K and 7.7 GPa (ref. 23). These agreements support the use of solid phases (silicate glass and crystalline alloys) as proxies for melts.

### Iron isotopic fractionation between Earth's mantle and core

**Figure 1:**
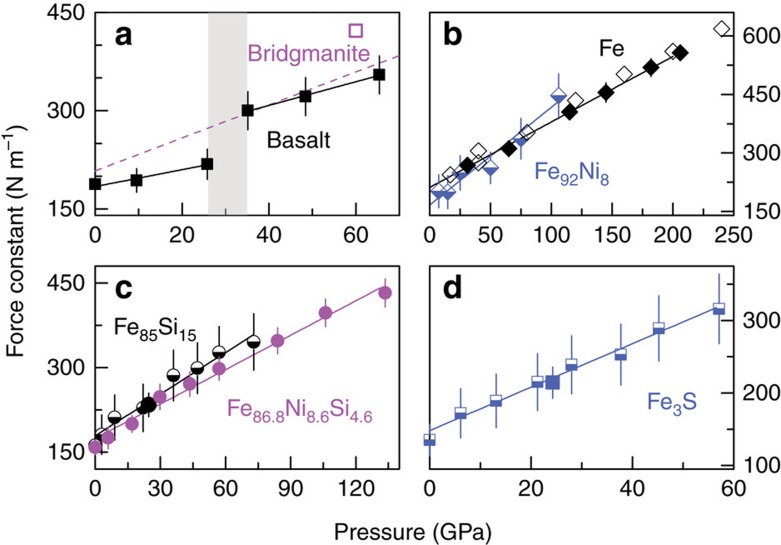
Force constants of iron bonds in basaltic glass and Fe and iron-rich alloys as a function of pressure. Black and blue solid squares, black solid diamonds, and black and magenta solid circles: basaltic glass (**a**), Fe (**b**), Fe_85_Si_15_ (**c**), Fe_86.8_Ni_8.6_Si_4.6_ (**c**) and Fe_3_S (**d**), respectively, from this study (Supplementary Table 2); half-filled diamonds, circles and squares: 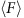 values for Fe_92_Ni_8_, Fe_85_Si_15_ and Fe_3_S evaluated based on the NRIXS data from Lin *et al*.^34^,^35^ using the SciPhon software; open square and diamonds: bridgmanite and Fe reported by Shahar *et al*.^25^; dashed line: bridgmanite reported by Rustad and Yin^56^. The error bars are 95% confidence intervals. Each high-pressure data point was measured at least 20 times and as many as 47 times. Solid lines: linear fits to the data for the basaltic glass, Fe and iron-rich alloys. Note the large shift in 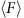 value of the basaltic glass (**a**) at ∼30 GPa, which corresponds to structural changes in the glass^29^,^37^.

The 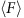 values of iron bonds of all investigated phases increase considerably with pressure (Fig. 1). The 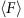 values of iron bonds in basaltic glass increase by ∼40% from 26 to 35 GPa (Fig. 1a), likely to be associated with the formation of tricluster oxygens^29^ and structural change^37^ in silicate glasses and melts. In contrast, no obvious discontinuity is observed in the 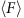 values of the alloys, which define linear trends with pressure (Fig. 1b–d). Even for Fe_86.8_Ni_8.6_Si_4.6_, no significant offset is measured when the alloy transitions from body-centred cubic to hexagonal close-packed at ∼20 GPa (Fig. 1c). The 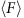 values of pure iron reported in the present study are identical within uncertainty to those of iron derived from NRIXS and theoretical calculations by Shahar *et al*.^25^ In details, however, the 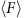 values calculated from a fit to their data are systematically greater than ours by ∼12 N m^−1^, which corresponds to a negligible difference of ∼0.003‰ in predicted *β*-factors at 3,500 K (Fig. 1b).

**Figure 2:**
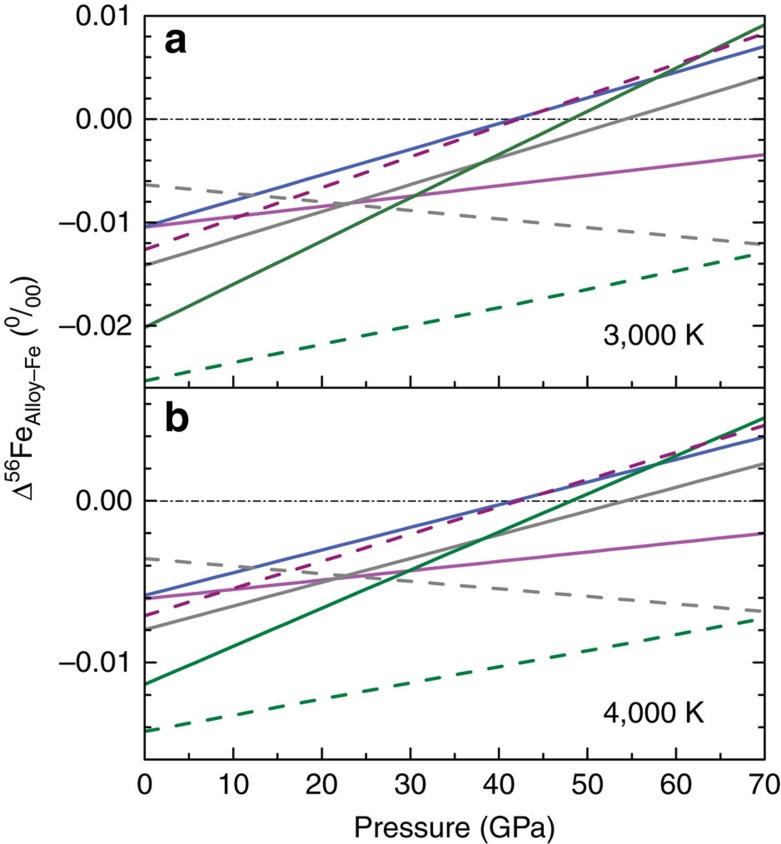
Comparisons of *β*-factors of iron-rich alloys at high pressure and temperature. Difference in the ^56^Fe/^54^Fe *β*-factors of iron-rich alloys with respect to pure iron at 3,000 K (**a**) and 4,000 K (**b**) as a function of pressure. Magenta, blue, grey and olive solid lines: Fe_86.8_Ni_8.6_Si_4.6_, Fe_85_Si_15_, Fe_92_Ni_8_ and Fe_3_S alloys, respectively, from this study. Purple, grey and olive dashed lines: FeO, Fe_3_C and FeH_*x*_, respectively, from Shahar *et al*.^25^ Δ^56^Fe_Alloy-Fe_=δ^56^Fe_Alloy_−δ^56^Fe_Fe_=1,000 × (ln β_Alloy_^56/54^Fe−ln β_Fe_^56/54^Fe). Black dash-dotted lines represent no iron isotopic fractionation between Fe-rich alloys and pure iron.

**Figure 3:**
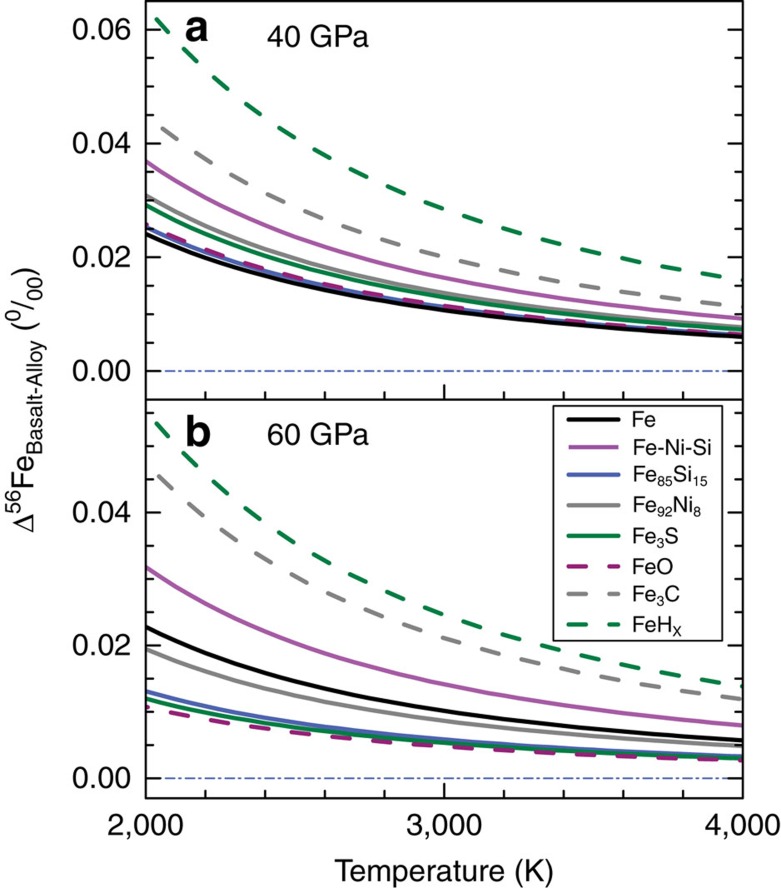
Equilibrium ^56^Fe/^54^Fe isotope fractionation between basaltic glass and iron-rich alloys at high pressure and temperature. **a**. Pressure at 40 GPa. **b**. Pressure at 60 GPa. Black, magenta, blue, grey and olive solid lines: Fe, Fe_86.8_Ni_8.6_Si_4.6_, Fe_85_Si_15_, Fe_92_Ni_8_, and Fe_3_S alloys, respectively, from this study. Purple, grey and olive dashed lines: FeO, Fe_3_C and FeH_*x*_, respectively, from Shahar *et al*.^25^ Δ^56^Fe_Basalt-Alloy_=δ^56^Fe_Basalt_−δ^56^Fe_Alloy_. Blue dash-dotted lines represent no iron isotopic fractionation between basaltic glass and Fe-rich alloys.

Linear fits to measured 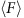 values versus *P* were calculated (Table 1), from which one can calculate the *β*-factor of basaltic glass, pure iron and Fe-rich alloy at any *P–T* conditions (Supplementary Figs 4 and 5). The quantity that we are interested in is the fractionation between metal and silicate under the conditions relevant to core formation conditions (∼40–60 GPa and 3,000–4,000 K for single-stage equilibration^45^,^46^,^47^). The silicate *β*-factor is given by the basaltic glass. For metal, the *β*-factor may be influenced by the composition of the alloy, which is shown in Figs 2 and 3 at a reference value of 3,500 K and variable pressures. Except for FeH_*x*_, the predicted 1,000 ln *β*-values of the alloys are within ∼±0.01‰ of the pure Fe value.

## Discussion

**Figure 4:**
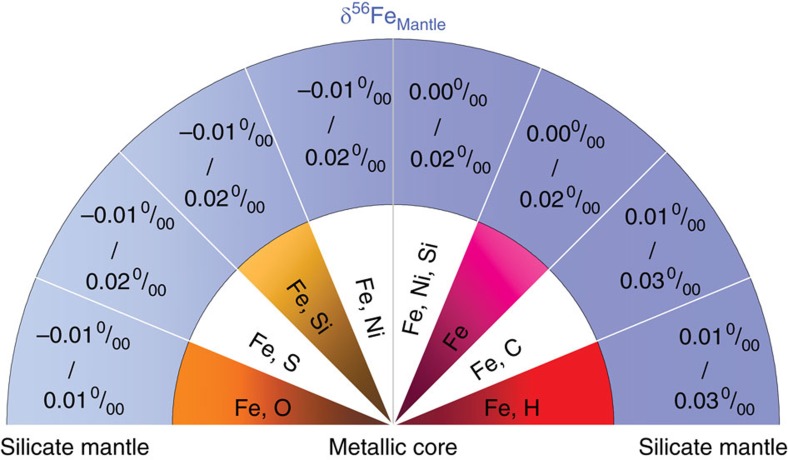
Predicted shifts in the iron isotopic composition of the silicate mantle due to core formation. Schematics for δ^56^Fe isotope signatures in the bulk silicate Earth (primitive mantle) with regard to varying compositional models of Earth's core. The segregating metal was assumed to equilibrate with the silicate mantle at the base of the magma ocean at ∼50 GPa and 3,500 K based on refs 45, 46, 47.

The temperature of metal-silicate equilibration in the early Earth is uncertain, because it is usually calculated from the pressure of equilibration assuming that Earth's mantle was close to the liquidus temperature of the silicate. The liquidus temperature itself is uncertain and it is possible that the silicate mantle was superheated in the aftermath of giant impacts, so that the temperatures were higher than those given by the liquidus^48^. Previous studies on the partitioning behaviour of moderately siderophile elements constrain the pressure–temperature conditions for high-pressure core formation in the Earth to be ∼40–60 GPa and ∼3,000–4,000 K, possibly accompanying deep magma oceans extending to >1,000 km depth^45^,^46^,^47^. In the Earth, ∼87% of the iron inventory is in the core, meaning that the silicate-metal equilibrium fractionation would be expressed in the mantle. Assuming a chondritic bulk Earth composition, the shift imposed on the mantle δ^56^Fe value by core formation would depend on the alloy composition (Fig. 4).

The theoretically derived iron *β*-factor of bridgmanite obtained by Shahar *et al*.^25^ are larger by ∼20% than the *β*-factor measured on the basaltic glass at ∼40–60 GPa. As a result, the fractionation values that they calculated were systematically heavier than ours by ∼0.01–0.02‰ at approximately 40–60 GPa and 3,500 K. For instance, for the Fe-C core model, the calculated δ^56^Fe_Mantle_ value is ∼+0.01–0.03‰ using basaltic glass data from the present study, whereas Shahar *et al*.^25^ calculated a δ^56^Fe_Mantle_ value of ∼+0.03–0.05‰ based on first-principles calculations of iron *β*-factor of bridgmanite. Although we agree with Shahar *et al*.^25^ that FeHx and Fe_3_C would lead to the largest shifts in δ^56^Fe values, we find that the shift would be smaller than what they predicted by 0.01–0.02‰.

Earth's core is unlikely to contain much H and C^27^,^28^ and the most probable alloying elements, in addition to Ni, are O, Si and S. Our results agree with the conclusion by Shahar *et al*.^25^ that FeO alloy would induce a negligible shift in the δ^56^Fe value of the mantle (Fig. 4). An important conclusion of the present work is that other alloys of Fe-S, Fe-Si and Fe-Ni-Si would also produce minimal isotopic fractionation in the silicate mantle relative to the bulk Earth. The inferred core-mantle Fe isotopic fraction at ∼40–60 GPa and ∼3,000–4,000 K for O, Si and S alloys is approximately five to ten times smaller than the observed Fe isotope composition for MORBs (δ^56^Fe of ∼+0.1‰ relative to chondrites)^6^. Our results thus indicate that high-pressure mantle-core equilibration cannot be responsible for the heavy Fe isotopic composition measured in basalts (Fig. 4). Therefore, our results reveal that the Fe isotopic composition of the mantle is representative of the bulk Earth and provides a baseline with which to compare the composition of extraterrestrial samples.

Our results also have some bearing on the iron isotopic compositions of rocks from Mars (SNC meteorites), Vesta (HED meteorites) and the angrite parent body. The *P–T* conditions of core formation in those planetary bodies are inferred to be ∼6 GPa–1,900 K for Mars^49^, ∼0.0001 GPa–1,873 K for Vesta^49^ and <0.5 GPa–2,173 K for the angrite parent body^50^. Under these conditions, we calculate that as on Earth, the equilibrium iron isotopic fractionation should be small (<0.02‰). Even if significant S is present in cores of these bodies, the results for Fe_3_S indicate that the fractionation would be minimal. This is consistent with the fact that SNC and HED meteorites have near-chondritic Fe isotopic compositions^2^,^15^. It does not explain, however, the heavy Fe isotopic composition of angrites (∼+0.1‰ relative to chondrites)^51^. In that case, other interpretations must be sought, which based on Si isotope systematics may involve isotopic fractionation during condensation in the solar nebula^52^ or impact-induced vaporization^53^. The emerging picture from this study supports the notion that core formation of planetary bodies in the Solar System is unlikely to fractionate significantly stable isotope ratios for elements such as Si, Cr and Fe. Other processes such as gas–solid fractionation in the nebula, impact-induced vaporization, or magma generation must be responsible for explaining the isotopic diversity of terrestrial and extraterrestrial rocks.

## Methods

### Sample synthesis

As the NRIXS technique is only sensitive to the Mössbauer isotope ^57^Fe, phases enriched in this isotope were synthesized in the laboratory from almost pure ^57^Fe-oxide and metal. The ^57^Fe-enriched tholeiitic basalt glass sample was synthesized from a mixture of SiO_2_, Al_2_O_3_, CaCO_3_, MgO, Na_2_CO_3_, K_2_CO_3_, TiO_2_ and Fe-enriched Fe_2_O_3_ in vertical gas mixing furnaces at the University of Lille and at CRPG-Nancy (France). The oxygen fugacity (*f*O_2_) was controlled using CO/CO_2_ gas mixtures. The chemical composition and homogeneity of the glass was examined using electron microprobe and Mössbauer spectroscopy. Its composition is Na_0.036_Ca_0.220_Mg_0.493_Fe_0.115_Al_0.307_Ti_0.012_K_0.002_Si_0.834_O_3_ with Fe^3+^/Fe_total_<0.02 (see ref. 20 for more details of the synthesis process). The pure Fe powder sample (with an enrichment of >95% ^57^Fe) was purchased from Cambridge Isotope Laboratories, Inc. The Fe-rich Fe_86.8_Ni_8.6_Si_4.6_ alloy was synthesized from a mixture of powder Fe, Ni and Si in an arc furnace in a pure Ar atmosphere at the Max Planck Institute for Solid State Research, Stuttgart, and Bayerisches Geoinstitut, University of Bayreuth^33^. The >95% ^57^Fe-enriched Fe_85_Si_15_ alloy was synthesized from a homogeneous mixture of Fe and Si by arc melting at the Advanced Photon Source, Argonne National Laboratory, USA^34^. The ∼65% ^57^Fe-enriched Fe_3_S sample was synthesized from the starting materials of ^57^Fe-enriched iron and troilite (FeS) in a multianvil apparatus at the Geophysical Laboratory, Carnegie Institution of Washington^35^.

### High-pressure synchrotron NRIXS experiments

We conducted *in situ* high-pressure NRIXS experiments on basaltic glass, Fe and Fe-rich alloys in DACs up to 206 GPa at sector 3ID-B of the Advanced Photon Source, Argonne National Laboratory (Supplementary Fig. 1). Each ^57^Fe-enriched sample was separately loaded into a sample chamber drilled into a beryllium gasket in a panoramic DAC. The starting samples of approximately 10–15 μm in thickness and 30 μm in diameter were loaded into panoramic DACs with culet sizes ranging from 50 to 400 μm in diameter, with Be gaskets of 3 mm in diameter and cubic boron nitride gasket inserts. For the basaltic glass, Fe and Fe-Ni-Si alloy, each NRIXS spectrum was scanned around the nuclear transition energy of ^57^Fe with a step size of 0.25 meV and a collection time of 5 s per energy step with an energy resolution of 1 meV. For the Fe-S and Fe–Si alloys, the scan step size was increased to 0.5 meV with an energy resolution of 2 meV, to increase the count rate for the inelastic peaks. Each NRIXS scan took about 1–1.5 h. Owing to the dilute Fe content in the basalt glass sample and the extended energy acquisition range, 20–47 NRIXS scans (approximately 2–3 days of beamtime) per pressure were collected and combined in order to achieve good statistics. All the NRIXS measurements were made at room temperature (∼300 K). A broad energy range (for example, from −110 to +140 meV for the basaltic glass) was scanned at high pressures, which is important to capture multiphonon contributions and possible high-energy vibration modes for the reliable determination of 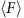 values^20^ (Supplementary Table 1). Pressure was calibrated using the ruby scale^54^ for the basaltic glass, Fe and Fe alloys below 70 GPa. Above 70 GPa, the Raman spectra of the diamond anvils were collected for use as a pressure gauge before and after each measurement^55^. The pressure was cross-checked with the previously characterized equation of state of Fe and Fe-Ni-Si alloy^33^.

### SciPhon software and data analysis

We applied a new approach based on moment calculations of NRIXS scattering spectra *S*(E) using the SciPhon software, as this method allows a better assessment of measurement uncertainties and potential systematic errors than using moments of the phonon density of states^32^,^41^. In the quasiharmonic lattice model, the third moment *R*_3_ of a measured NRIXS spectrum *S*(*E*) is used to calculate the mean force constant 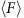 of iron bonds in the samples (in N/m)^32^,^41^:


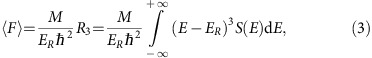


where *M* is the mass of the nuclear resonant isotope (^57^Fe in this study), *E* is the energy difference between incident X-ray and the nuclear resonance *E*_0_ (in meV) and 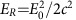 is the free recoil energy (that is, 1.956 meV for the *E*_0_=14.4125, keV nuclear transition of ^57^Fe). Within the harmonic approximation, the *β*-factors and the equilibrium isotope fractionation between two phases A and B at high temperature (>500 K; ref. 32) can be calculated from 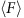 given above using the following relations^20^,^32^:


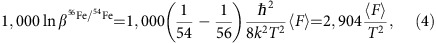


and


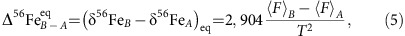


where 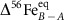 is the permil difference in isotopic ratios (^56^Fe/^54^Fe) of phases *A* and *B* at equilibrium (that is, if those phases were juxtaposed and the isotopes of iron partitioned between them according to the laws of equilibrium thermodynamics), *k* is Boltzmann's constant, *ħ* is the reduced Planck constant and *T* is temperature. The mean force constants <*F*> of iron bonds in the samples were calculated from NRIXS spectra using the SciPhon software, which includes a correction for non-constant baseline. The uncertainties on the force constant measurements (95% confidence interval, comprising both systematic and random errors) are ∼5–10%. The <*F*> values from SciPhon agree with PHOENIX but those from PHOENIX without baseline subtraction appear more scattered (Supplementary Figs 2 and 3). Several parameters that can be calculated from NRIXS data by using SciPhon, have been compiled in Supplementary Table 2, including estimates of the Lamb–Mössbauer factor, kinetic energy per atom, force constant, internal energy, vibrational specific heat, vibrational entropy, critical temperature, sound velocities (Debye, compressional-wave and shear-wave) and coefficients of the polynomial used to calculate *β*-factors at any temperature.

### Data availability

The data sets generated during and/or analysed during the current study are available as Supplementary Information and from the corresponding authors.

## Additional information

**How to cite this article:** Liu, J. *et al*. Iron isotopic fractionation between silicate mantle and metallic core at high pressure. *Nat. Commun.* **8,** 14377 doi: 10.1038/ncomms14377 (2017).

**Publisher's note**: Springer Nature remains neutral with regard to jurisdictional claims in published maps and institutional affiliations.

## Supplementary Material

Supplementary Information Supplementary Figures and Supplementary Tables

Supplementary Data 1 Supplementary Dataset for Supplementary Figures 6-8

## Figures and Tables

**Table 1:** Pressure dependence of the force constant 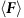 values of iron bonds in basaltic glass and Fe-rich alloys.

**Sample**	**Pressure range (GPa)**	***a*** **(N** **m**^**−1**^ **GPa**^**−1**^**)**	***b*** **(N** **m**^**−1**^**)**	***R***^**2**^
Basaltic glass	0–26	1.29 (±0.24)	184.1 (±4.5)	0.929
	35–65	1.81 (±0.09)	235.8 (±5.1)	0.994
Fe	0–206	1.68 (±0.04)	211.7 (±4.7)	0.998
Fe_86.8_Ni_8.6_Si_4.6_	0–133	2.03 (±0.09)	174.2 (±6.5)	0.986
Fe_92_Ni_8_*	8–106	2.50 (±0.26)	167.0 (±17.3)	0.950
Fe_85_Si_15_*	0–70	2.46 (±0.19)	178.9 (±7.8)	0.960
Fe_3_S*	0–57	2.99 (±0.21)	148.0 (±6.9)	0.966

The linear relationships between the force constant 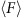 and pressure (*P*) for those samples were expressed as 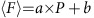, with 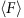 in the unit of N/m and *P* in the unit of GPa.

^*^The 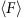 values of iron bonds in Fe_92_Ni_8_, Fe_85_Si_15_, and Fe_3_S were calculated from this study and NRIXS data from Lin *et al*.^34^,^35^. The <*F*> values for Fe_85_Si_15_ and Fe_3_S (corresponding to solid symbols in Fig. 1c,d) at ∼24 GPa from this study are in agreement with those (corresponding to half-filled symbols in Fig. 1c,d) calculated by using the SciPhon software with previous NRIXS data, demonstrating the reproducibility of NRIXS results.
