# Supplementary material for: Iron isotopic fractionation between silicate mantle and metallic core at high pressure
Source: Nat Commun. 2017 Feb 20;8:14377. doi: 10.1038/ncomms14377 (PMC5321738; doi:10.1038/ncomms14377)
Supplement: Supplementary Information — Supplementary Figures and Supplementary Tables [file ncomms14377-s1.pdf]

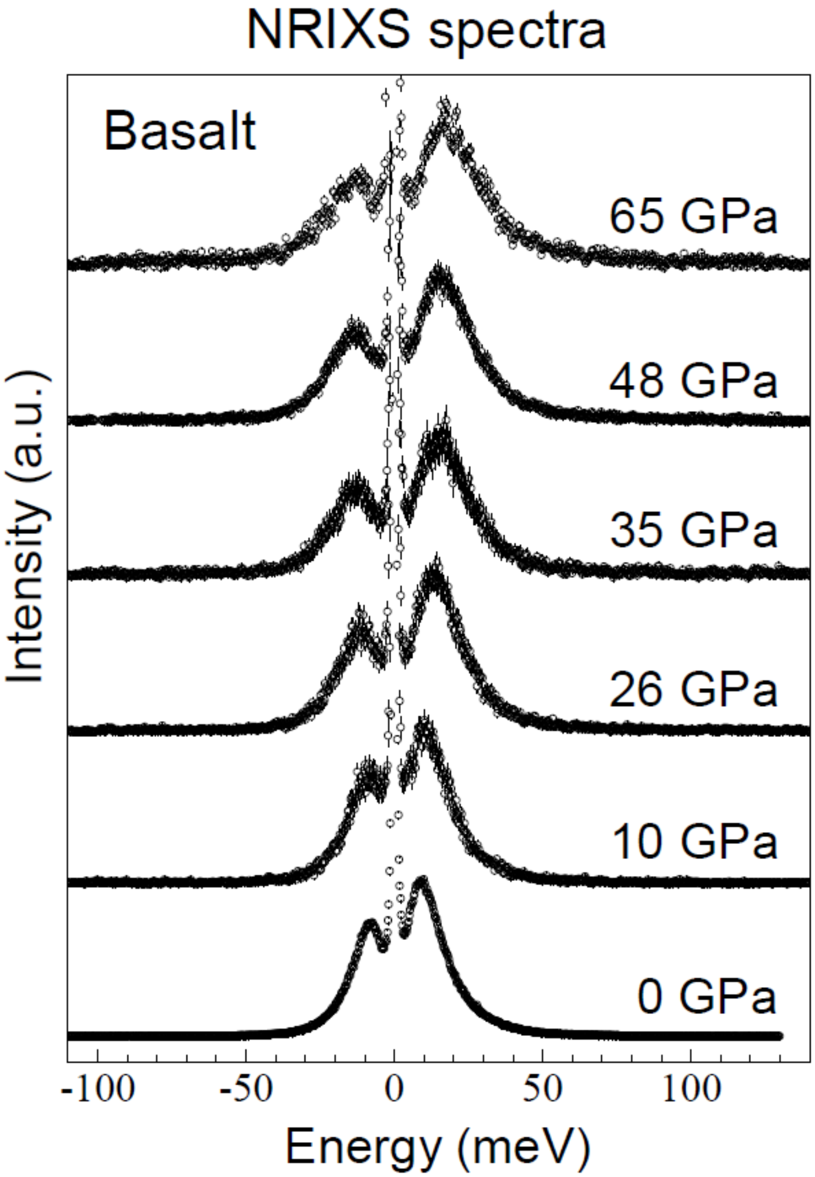

Supplementary Figure 1 Representative NRIXS spectra of the basaltic glass and Fe-rich alloys at high pressures. The central peaks at 0 meV correspond to the elastic scattering. Open symbols: experimental data; vertical ticks: statistical errors.

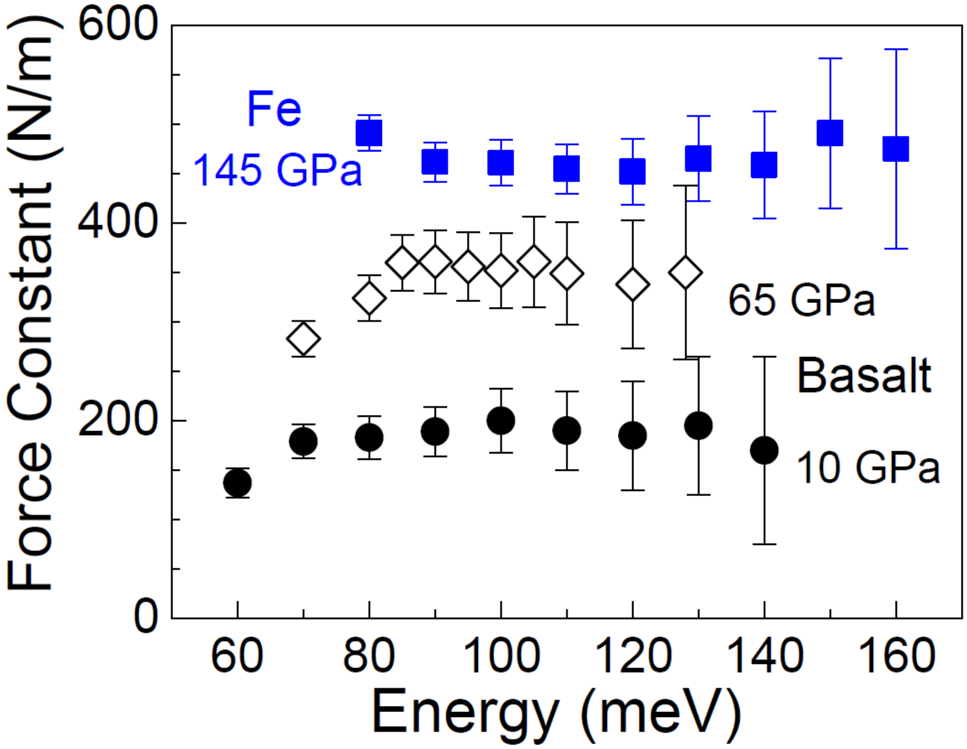

Supplementary Figure 2 The force constants <*F*> of basaltic glass and Fe determined by SciPhon as a function of the energy range fitted in the analysis at high pressures. The uncertainties increase with the fitted energy range, but the calculated <*F*> values fall in a narrow range when the fitted energy range is greater than ~90 meV.

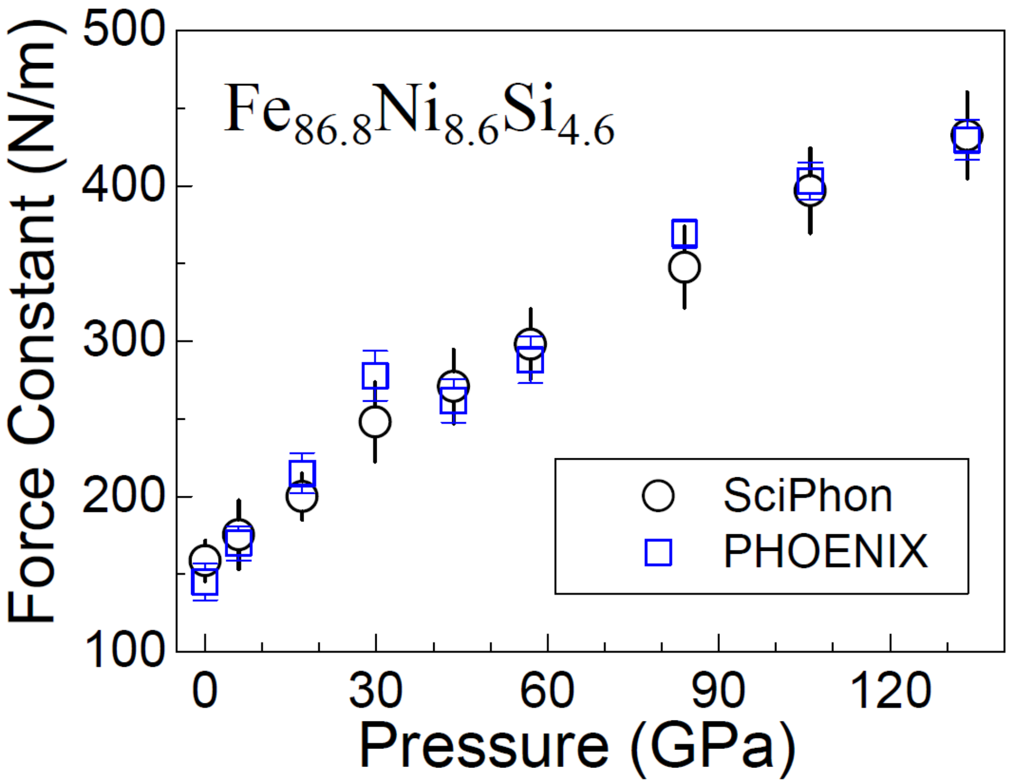

Supplementary Figure 3 The mean force constants <*F*> of bcc- and hcp-$Fe_{86.8}Ni_{8.6}Si_{4.6}$ alloy at high pressures.

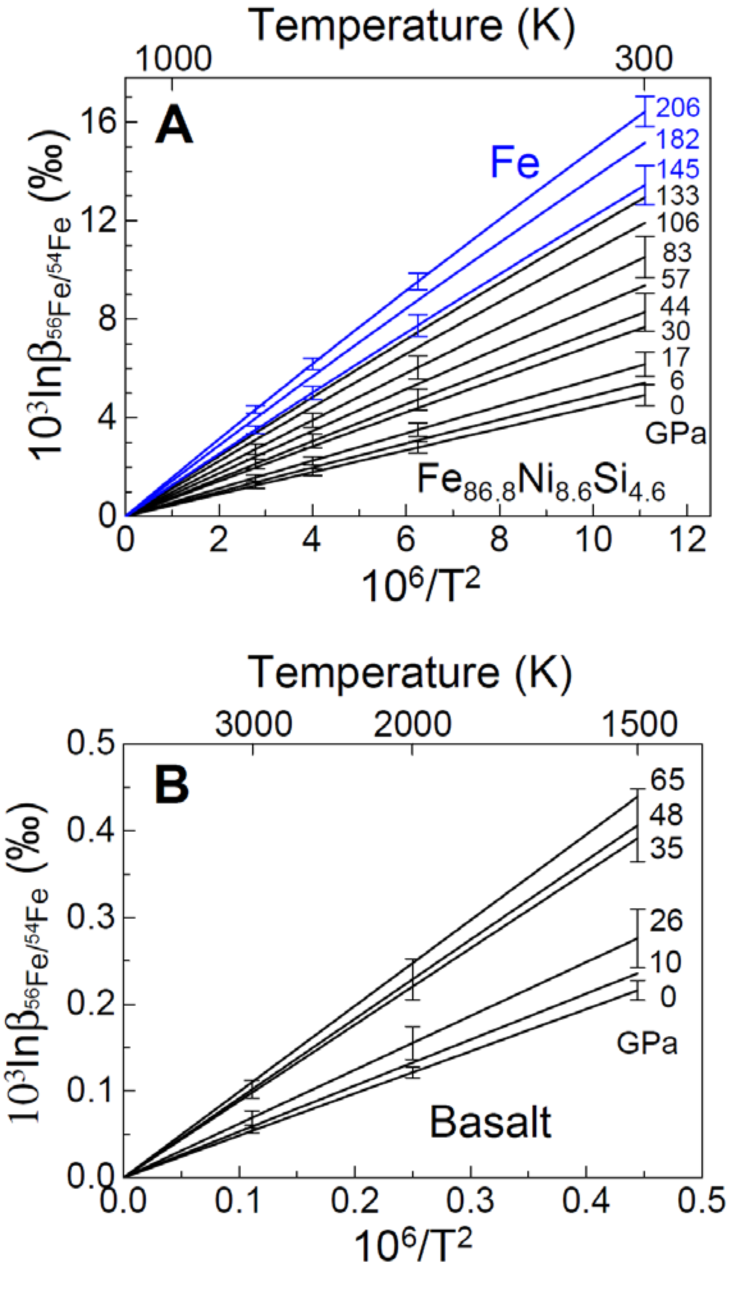

Supplementary Figure 4 Temperature dependence of the $^{56}Fe/^{54}Fe$ *β*-factors for Fe and $Fe_{86.8}Ni_{8.6}Si_{4.6}$ alloy (***A***) and the basaltic glass (***B***) at high pressures. The $^{56}Fe/^{54}Fe$ *β*-factors and error bars are computed using the SciPhon software.

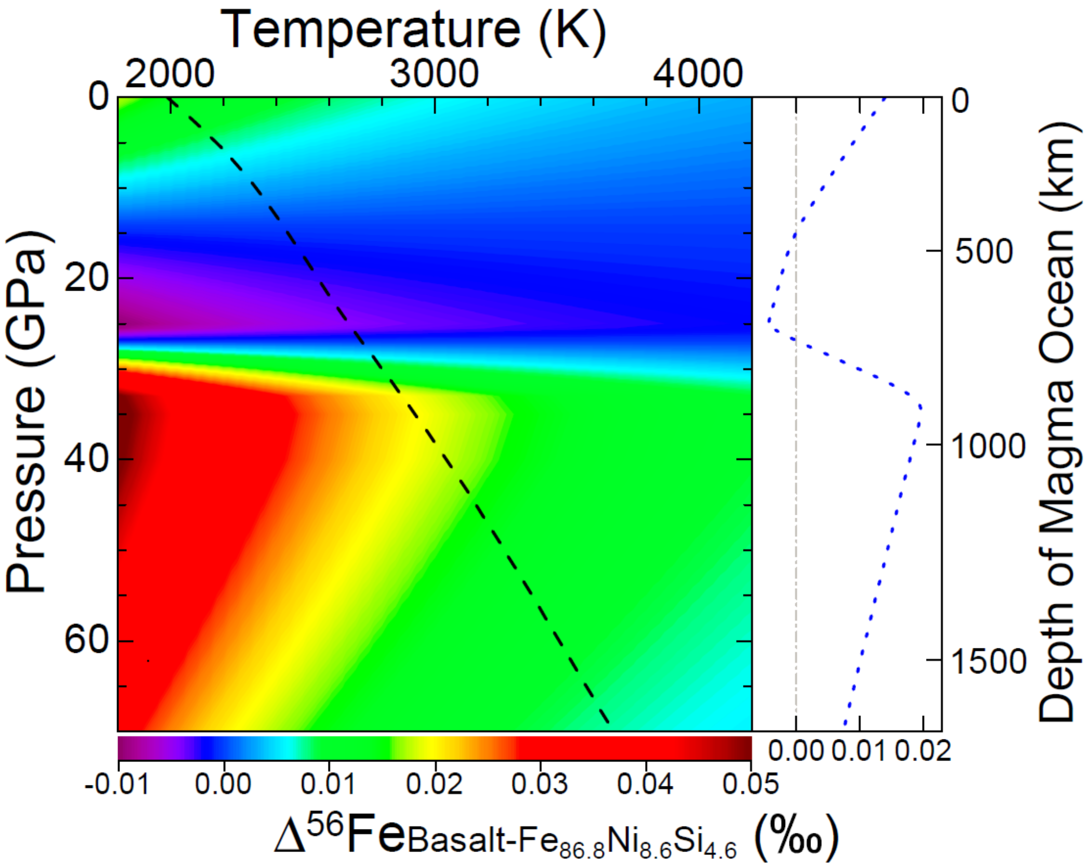

Supplementary Figure 5 Equilibrium $^{56}Fe/^{54}Fe$ isotope fractionation between basaltic glass and $Fe_{86.8}Ni_{8.6}Si_{4.6}$ alloy at high *P*-*T* conditions with regard to varying depth of magma ocean. Black dashed line: example of metal-silicate equilibration temperatures as a function of pressure corresponding to the mantle liquidus; blue dotted line: iron isotopic fractionation along the mantle liquidus with the varying depth of magma ocean; dash-dotted lines represent no iron isotopic fractionation between basaltic glass and $Fe_{86.8}Ni_{8.6}Si_{4.6}$ alloy. Two separate linear regressions were used for regressing the force constant of iron bounds in basaltic glass below and above 26 GPa.

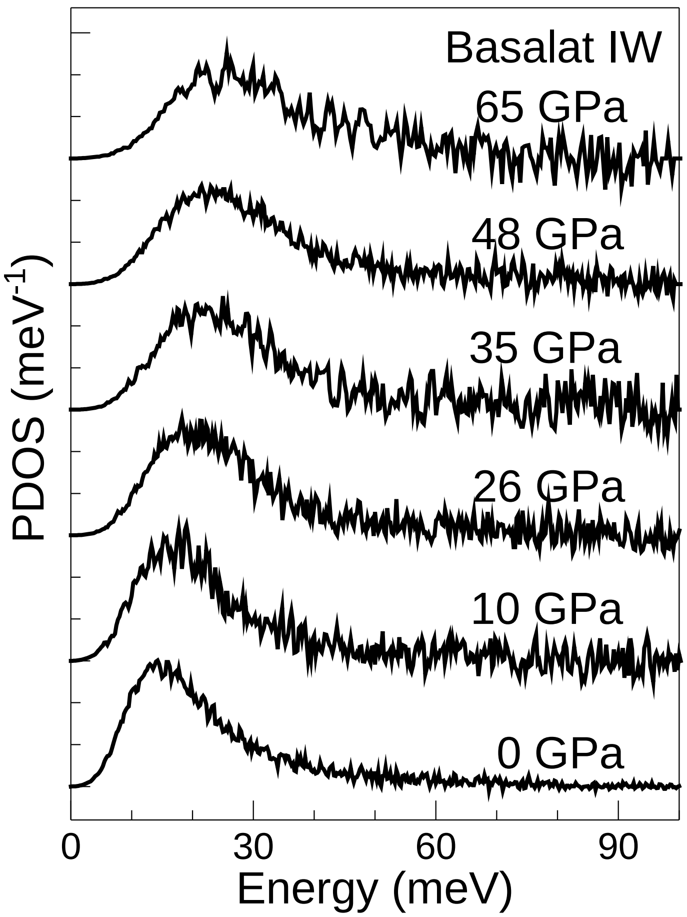

Supplementary Figure 6 Phonon density of states (PDOS) of the basaltic glass from high-pressure NRIXS measurements using SciPhon.

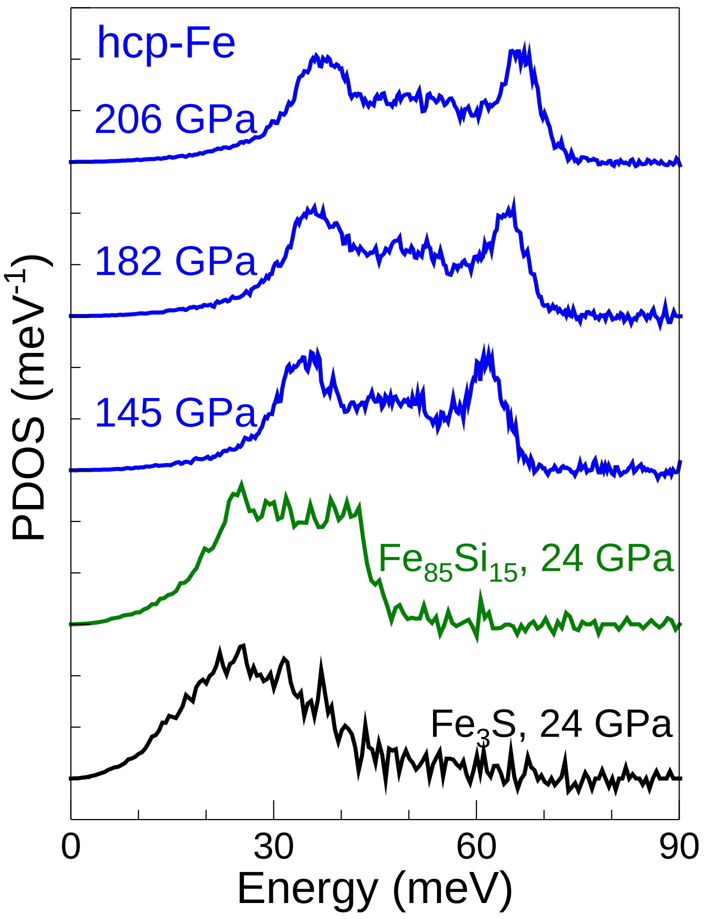

Supplementary Figure 7 Phonon density of states (PDOS) of hcp-Fe, Fe_3S, and $Fe_{85}Si_{15}$ from high-pressure NRIXS measurements using SciPhon.

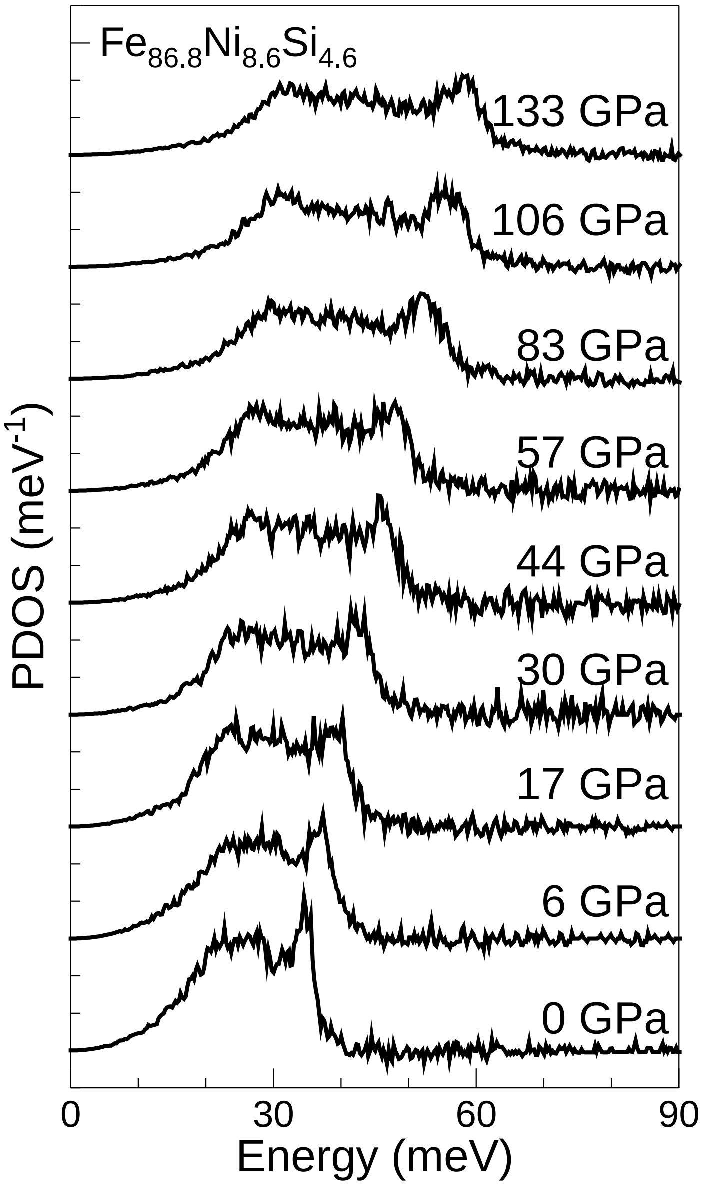

Supplementary Figure 8 Phonon density of states (PDOS) of $Fe_{86.8}Ni_{8.6}Si_{4.6}$ alloy from high-pressure NRIXS measurements using SciPhon.

Supplementary Table 1 Experimental parameters for our NRIXS data collection.

Sample	Pressure (GPa)	Energy Range (meV)[¶]	Number of Scans[§]	Scan Time (sec)[†]
Basaltic glass	0.0001	-130 to +130	8	24
Basaltic glass	10	-130 to +170	20	80
Basaltic glass	26	-100 to +140	20	80
Basaltic glass	35	-100 to +140	29	110
Basaltic glass	48	-110 to +140	45	180
Basaltic glass	65	-110 to +150	47	185
Fe	145	-110 to +180	25	125
Fe	182	-110 to +140	28	140
Fe	206	-110 to +140	31	155
$Fe_{86.8}Ni_{8.6}Si_{4.6}$	0.0001	-110 to +110	3	10
$Fe_{86.8}Ni_{8.6}Si_{4.6}$	30	-110 to +110	29	98
$Fe_{86.8}Ni_{8.6}Si_{4.6}$	44	-110 to +110	29	105
$Fe_{86.8}Ni_{8.6}Si_{4.6}$	57	-110 to +110	25	115
$Fe_{86.8}Ni_{8.6}Si_{4.6}$	83	-110 to +110	20	100
$Fe_{86.8}Ni_{8.6}Si_{4.6}$	106	-110 to +120	20	100
$Fe_{86.8}Ni_{8.6}Si_{4.6}$	133	-110 to +120	20	100
$Fe_{85}Si_{15}$	24	-100 to +110	31	155
Fe_3S	24	-100 to +110	27	135

[¶]The energy range over which NRIXS data was collected, relative to the nuclear transition energy of ^{57}Fe (14.4125 keV). For the Fe_3S and $Fe_{85}Si_{15}$ alloys, the scan step size was 0.5 meV with an energy resolution of 2 meV. For other samples, the scan step size was 0.25 meV with an energy resolution of 1 meV.

[§]It reflects the total number of scans at each energy step.

[†]It reflects the total seconds counted at each energy step.

Supplementary Table 2 Thermoelastic properties derived from NRIXS data.

	Basaltic glass					
Pressure (GPa)	0.0001	9.5 ± 0.3	25.8 ± 0.6	35.1 ± 0.7	48.4 ± 1.2	65.4 ± 1.6
Temperature from detailed balance (K):	308.0	273.6	287.1	285.7	304.2	249.4
**Parameters from S**						
Lamb-Mössbauer factor from S	0.5651 ± 0.0023	0.6098 ± 0.0079	0.7121 ± 0.0065	0.7560 ± 0.0050	0.7718 ± 0.0045	0.8065 ± 0.0045
Mean square displacement $\langle z^2\rangle$ from S (Å^2)	0.01071 ± 0.00007	0.00928 ± 0.00023	0.00637 ± 0.00016	0.00525 ± 0.00013	0.00486 ± 0.00011	0.00404 ± 0.00012
Internal energy/atom from S (meV)	28.54 ± 0.65	28.37 ± 1.32	28.73 ± 1.19	30.97 ± 1.57	31.07 ± 1.37	30.92 ± 1.76
Kinetic energy/atom from S (meV)	14.27 ± 0.33	14.18 ± 0.66	14.37 ± 0.60	15.49 ± 0.78	15.54 ± 0.69	15.46 ± 0.88
Force constant $\langle F\rangle$ from S (N/m)	187.9 ± 9.1	193.3 ± 20.0	218.3 ± 26.4	300.1 ± 29.6	321.5 ± 33.1	354.7 ± 32.4
$^{56}Fe/^{54}Fe$ β coefficients from S						
$1000 \ln \beta = A_1/T^2 + A_2/T^4 + A_3/T^6$ (T in K)						
A_1	487045 ± 25835	531800 ± 89153	622806 ± 75323	883840 ± 103139	917433 ± 94375	992225 ± 113249
A_2	-2499859579 ± 362830903	-2767067429 ± 1111830911	-3278961493 ± 877251096	-6604017352 ± 1349982531	-6379030730 ± 1234995617	-6577866750 ± 1461981888
A_3	34037320533882 ± 9397226860024	32472515323750 ± 23534087360276	37048436253731 ± 17371347871280	97622780253959 ± 29600979463874	83879858886861 ± 27145185127542	75852479785072 ± 31707359296823
$1000 \ln \beta = B_1\langle F\rangle/T^2 - B_1\langle F\rangle^2/T^4$ (T in K)						
B_1	2853.4	2853.4	2853.4	2853.4	2853.4	2853.4
B_2	73186.6	69566.1	60428.8	57843.2	52944.2	47624.9
**Parameters from g**						
Lamb-Mössbauer factor from g	0.5627	0.6081	0.7106	0.7554	0.7713	0.8048
Mean square displacement $\langle z^2\rangle$ from g (Å^2)	0.010790	0.009334	0.006412	0.005263	0.004873	0.004076
$d\langle z^2\rangle/dT$ (Å^2/K)	0.00003442	0.00002959	0.00001985	0.00001596	0.00001467	0.00001208
Critical temperature (K):	545.3	634.3	945.2	1175.9	1279.2	1554.1
Resilience (N/m):	40.12	46.66	69.54	86.48	94.11	114.3
Internal energy/atom from g (meV)	29.11	28.98	29.45	31.74	31.85	31.74
Kinetic energy/atom from g (meV)	14.56	14.49	14.72	15.87	15.93	15.87
Vibrational entropy (kb/atom)	1.367	1.280	1.151	1.088	1.045	0.945
Helmholtz free energy (meV)	-6.234	-4.098	-0.310	3.612	4.846	7.327
Vibrational specific heat (kb/atom)	0.9519	0.9300	0.9157	0.9251	0.9154	0.8818
Lamb-Mössbauer factor at T=0 from g	0.8845	0.8941	0.9093	0.9155	0.9191	0.9273
Kinetic energy/atom at T=0 from g (meV)	5.977	6.289	7.005	8.373	8.641	9.110
Force constant from g (N/m)	167.7	184.7	216.7	306.0	317.6	345.4
$^{56}Fe/^{54}Fe$ β coefficients from g						
$1000 \ln \beta = A_1/T^2 + A_2/T^4 + A_3/T^6$ (T in K)						
A_1	478517	526915	618314	873225	906297	985619
A_2	-2474782640	-2777684039	-3317972347	-6819420085	-6536563587	-6739161275
A_3	34675991241897	35418631015657	42528329767727	121725877905789	103724390199225	95095457934447
Velocities from g						
Input density (g/cc)	2.75	3.05	3.36	3.51	3.65	3.83
Input bulk modulus (GPa)	79	120	215	265	300	350
Debye velocity (m/s)	3422 ± 94	3326 ± 173	3753 ± 143	4778 ± 155	4845 ± 185	4949 ± 155
p-wave velocity (m/s)	6408 ± 57	7132 ± 96	8864 ± 75	9966 ± 87	10330 ± 103	10810 ± 85
s-wave velocity (m/s)	3042 ± 89	2939 ± 165	3307 ± 136	4226 ± 147	4282 ± 176	4370 ± 147
Poisson ratio:	0.355	0.398	0.419	0.390	0.396	0.402

Supplementary Table 2 Continued.

	$Fe_{86.8}Ni_{8.6}Si_{4.6}$					
Pressure (GPa)	0.0001	5.9 ± 0.2	16.5 ± 0.4	29.8 ± 0.9	44.4 ± 1.1	57.2 ± 1.4
Temperature from detailed balance (K):	293.8	281.9	284.4	260.2	258.6	261.7
**Parameters from S**						
Lamb-Mössbauer factor from S	0.7520 ± 0.0050	0.7857 ± 0.0055	0.8048 ± 0.0046	0.8385 ± 0.0039	0.8506 ± 0.0039	0.8625 ± 0.0036
Mean square displacement $<z^2>$ from S (Å^2)	0.00535 ± 0.00012	0.00453 ± 0.00016	0.00408 ± 0.00012	0.00331 ± 0.00010	0.00304 ± 0.00010	0.00278 ± 0.00008
Internal energy/atom from S (meV)	28.24 ± 0.83	27.51 ± 1.32	28.29 ± 0.92	28.61 ± 1.35	29.47 ± 1.22	29.83 ± 1.12
Kinetic energy/atom from S (meV)	14.12 ± 0.41	13.75 ± 0.66	14.15 ± 0.46	14.31 ± 0.68	14.74 ± 0.61	14.92 ± 0.56
Force constant $<F>$ from S (N/m)	158.6 ± 13.2	175.3 ± 22.2	199.9 ± 15.1	248.1 ± 25.6	270.8 ± 23.9	298.1 ± 22.7
$^{56}Fe/^{54}Fe$ β coefficients from S						
$1000 \ln \beta = A_1/T^2 + A_2/T^4 + A_3/T^6$ (T in K)						
A_1	452543 ± 37689	500101 ± 63283	570365 ± 43204	719377 ± 81629	772589 ± 68435	880340 ± 64837
A_2	-855972534 ± 382195770	-1090130245 ± 475271103	-1363620558 ± 297332113	-2672240644 ± 812264625	-2427052410 ± 591330677	-3516512957 ± 608821645
A_3	2163092347903 ± 7521159222783	2238583565501 ± 6279824759880	2019393441627 ± 3723379960435	16891344788688 ± 13915961179875	5211811252884 ± 9071118496761	16616074255008 ± 10152093922560
$1000 \ln \beta = B_1<F>/T^2 - B_1<F>^2/T^4$ (T in K)						
B_1	2853.4	2853.4	2853.4	2853.4	2853.4	2853.4
B_2	33102.4	34702.4	33583.2	39173.1	32339.1	35058.9
**Parameters from g**						
Lamb-Mössbauer factor from g	0.7514	0.7844	0.8032	0.8371	0.8503	0.8625
Mean square displacement $<z^2>$ from g (Å^2)	0.005363	0.004557	0.004113	0.003336	0.003043	0.002776
$d<z^2>/dT$ (Å^2/K)	0.00001631	0.00001370	0.00001220	0.00000964	0.00000862	0.00000775
Critical temperature (K):	1150.7	1370.1	1538.3	1945.8	2176.9	2423.3
Resilience (N/m):	84.66	100.8	113.1	143.2	1.60.1	178.2
Internal energy/atom from g (meV)	28.97	28.28	29.10	29.44	30.32	30.70
Kinetic energy/atom from g (meV)	14.49	14.14	14.55	14.72	15.16	15.35
Vibrational entropy (kb/atom)	1.148	1.039	1.008	0.904	0.882	0.831
Helmholtz free energy (meV)	-0.702	1.426	3.047	6.085	7.532	9.216
Vibrational specific heat (kb/atom)	0.9500	0.9031	0.9099	0.8722	0.8857	0.8646
Lamb-Mössbauer factor at T=0 from g	0.9137	0.9223	0.9252	0.9330	0.9349	0.9383
Kinetic energy/atom at T=0 from g (meV)	6.548	6.813	7.328	8.081	8.575	9.070
Force constant from g (N/m)	155.5	175.2	199.2	252.1	269.1	307.9
$^{56}Fe/^{54}Fe$ β coefficients from g						
$1000 \ln \beta = A_1/T^2 + A_2/T^4 + A_3/T^6$ (T in K)						
A_1	443667	499804	568313	719236	767989	878600
A_2	-822858307	-1085959414	-1401612555	-2703060703	-2452598552	-3612963004
A_3	2384925975050	3255968870400	4989288511131	21210514588007	9623131674342	25675744782979
Velocities from g						
Input density (g/cc)	7.705	7.957	8.377	9.157	9.540	9.865
Input bulk modulus (GPa)	163	190	235	300	370	440
Debye velocity (m/s)	3146 ± 54	3346 ± 68	3523 ± 61	3930 ± 101	4080 ± 128	4245 ± 84
p-wave velocity (m/s)	5680 ± 36	5978 ± 46	6416 ± 38	7009 ± 64	7507 ± 79	7973 ± 213
s-wave velocity (m/s)	2818 ± 51	2982 ± 64	3136 ± 58	3503 ± 96	3630 ± 121	3772 ± 81
Poisson ratio:	0.290	0.334	0.343	0.334	0.347	0.356

Supplementary Table 2 Continued.

	$Fe_{86.8}Ni_{8.6}Si_{4.6}$			Fe		
Pressure (GPa)	83 ± 2	106 ± 2	133 ± 2	145 ± 3	182 ± 5	206 ± 6
Temperature from detailed balance (K):	274.9	281.6	296.8	284.1	279.6	286.4
**Parameters from S**						
Lamb-Mössbauer factor from S	0.8820 ± 0.0030	0.8897 ± 0.0029	0.8943 ± 0.0024	0.9131 ± 0.0018	0.9195 ± 0.0015	0.9226 ± 0.0013
Mean square displacement $<z^2>$ from S (Å^2)	0.00236 ± 0.00007	0.00219 ± 0.00007	0.00210 ± 0.00006	0.00171 ± 0.00004	0.00158 ± 0.00004	0.00151 ± 0.00003
Internal energy/atom from S (meV)	29.96 ± 1.15	30.80 ± 1.13	31.65 ± 1.12	31.28 ± 0.93	32.34 ± 0.95	33.10 ± 0.78
Kinetic energy/atom from S (meV)	14.98 ± 0.57	15.40 ± 0.57	15.83 ± 0.56	15.64 ± 0.46	16.17 ± 0.48	16.55 ± 0.39
Force constant $<F>$ from S (N/m)	347.6 ± 26.1	396.8 ± 27.5	432.5 ± 27.9	455.5 ± 24.6	519.0 ± 22.6	556.8 ± 19.1
$^{56}Fe/^{54}Fe$ β coefficients from S						
1000 ln β = $A_1/T^2 + A_2/T^4 + A_3/T^6$ (T in K)						
A_1	991927 ± 74370	1132319 ± 78425	1234114 ± 79486	1279730 ± 70047	1480916 ± 64577	1588711 ± 54479
A_2	-4216363469 ± 891020969	-5862610202 ± 995909867	-6539594439 ± 1108528014	-6451244752 ± 1138807721	-9812369629 ± 830672017	-10967069895 ± 681878990
A_3	18283263766495 ± 18765311474413	41211258369178 ± 22302386417286	39315685892788 ± 27037391424976	16002379437505 ± 33637922607805	83832202412996 ± 18605221856532	94771605498907 ± 14918563186621
1000 ln β = $B_1<F>/T^2 - B_1<F>^2/T^4$ (T in K)						
B_1	2853.4	2853.4	2853.4	2853.4	2853.4	2853.4
B_2	33257.1	34403.2	32690.4	31214.1	33067.6	32076.3
**Parameters from g**						
Lamb-Mössbauer factor from g	0.8818	0.8897	0.8937	0.9131	0.9195	0.9222
Mean square displacement $<z^2>$ from g (Å^2)	0.002360	0.002194	0.002108	0.001706	0.001576	0.001520
$d<z^2>/dT$ (Å^2/K)	0.00000640	0.00000584	0.00000555	0.00000425	0.00000382	0.00000363
Critical temperature (K):	2933.2	3210.7	3380.2	4418.2	4912.1	5168.9
Resilience (N/m):	215.8	236.22	248.7	325.0	361.3	380.3
Internal energy/atom from g (meV)	30.83	31.68	32.53	32.18	33.27	34.03
Kinetic energy/atom from g (meV)	15.42	15.84	16.27	16.09	16.64	17.01
Vibrational entropy (kb/atom)	0.7507	0.7156	0.6947	0.6320	0.5960	0.5776
Helmholtz free energy (meV)	11.43	13.18	14.57	15.84	17.86	19.10
Vibrational specific heat (kb/atom)	0.8301	0.8188	0.8182	0.7857	0.7686	0.7644
Lamb-Mössbauer factor at T=0 from g	0.9438	0.9460	0.9471	0.9518	0.9538	0.9547
Kinetic energy/atom at T=0 from g (meV)	9.618	10.252	10.789	11.001	11.778	12.267
Force constant from g (N/m)	347.1	396.0	430.0	447.6	519.6	556.2
$^{56}Fe/^{54}Fe$ β coefficients from g						
1000 ln β = $A_1/T^2 + A_2/T^4 + A_3/T^6$ (T in K)						
A_1	990350	1129919	1227060	1277132	1482542	1587030
A_2	-4202140113	-5889817709	-6487299080	-6361250530	-10040066520	-11149521537
A_3	21957997504812	49902621168849	44822737751239	15813392696196	111690071429084	123201152115770
Velocities from g						
Input density (g/cc)	10.42	10.84	11.28	12.04	12.30	12.89
Input bulk modulus (GPa)	565	670	815	880	970	1160
Debye velocity (m/s)	4549 ± 89	4614 ± 69	4636 ± 67	5641 ± 90	5931 ± 102	5812 ± 80
p-wave velocity (m/s)	8718 ± 53	9171 ± 40	9729 ± 38	10330 ± 202	10770 ± 218	11202 ± 221
s-wave velocity (m/s)	4039 ± 85	4090 ± 65	4100 ± 64	5021 ± 86	5281 ± 98	5159 ± 77
Poisson ratio:	0.363	0.376	0.392	0.345	0.342	0.365

Supplementary Table 2 Continued.

	$Fe_{85}Si_{15}$	Fe_3S
Pressure (GPa)	24.4 ± 0.9	24.0 ± 0.9
Temperature from detailed balance (K):	294.9	276.7
**Parameters from S**		
Lamb-Mössbauer factor from S	0.8161 ± 0.0059	0.7597 ± 0.0076
Mean square displacement $<z^2>$ from S (Å^2)	0.00381 ± 0.00013	0.00516 ± 0.00018
Internal energy/atom from S (meV)	29.13 ± 1.10	28.38 ± 1.29
Kinetic energy/atom from S (meV)	14.56 ± 0.55	14.19 ± 0.64
Force constant $<F>$ from S (N/m)	233.8 ± 20.6	214.2 ± 25.7
$^{56}Fe/^{54}Fe$ β coefficients from S		
$1000 \ln \beta = A_1/T^2 + A_2/T^4 + A_3/T^6$ (T in K)		
A_1	667065 ± 58689	611129 ± 73337
A_2	-1976006890 ± 510139781	-2136152467 ± 647675781
A_3	6981558744210 ± 7922442915825	11606862101993 ± 10047243487594
$1000 \ln \beta = B_1<F>/T^2 - B_1<F>^2/T^4$ (T in K)		
B_1	2853.4	2853.4
B_2	34777.0	43836
**Parameters from g**		
Lamb-Mössbauer factor from g	0.8097	0.7554
Mean square displacement $<z^2>$ from g (Å^2)	0.003961	0.005264
$d<z^2>/dT$ (Å^2/K)	0.00001185	0.00001616
Critical temperature (K):	1583.5	1161.2
Resilience (N/m):	116.5	85.4
Internal energy/atom from g (meV)	29.94	29.14
Kinetic energy/atom from g (meV)	14.97	14.57
Vibrational entropy (kb/atom)	0.9712	1.0436
Helmholtz free energy (meV)	4.83	2.16
Vibrational specific heat (kb/atom)	0.9097	0.9002
Lamb-Mössbauer factor at T=0 from g	0.9277	0.9196
Kinetic energy/atom at T=0 from g (meV)	7.914	7.29
Force constant from g (N/m)	231.3	213.5
$^{56}Fe/^{54}Fe$ β coefficients from g		
$1000 \ln \beta = A_1/T^2 + A_2/T^4 + A_3/T^6$ (T in K)		
A_1	660128	609216
A_2	-1970292046	-2162540081
A_3	9646711326442	14898422858483
Velocities from g		
Input density (g/cc)	8.50	7.98
Input bulk modulus (GPa)	275	280
Debye velocity (m/s)	3583 ± 91	2930 ± 54
p-wave velocity (m/s)	6772 ± 152	6633 ± 195
s-wave velocity (m/s)	3183 ± 87	2585 ± 52
Poisson ratio:	0.358	0.410
